# Recreating Human Skin In Vitro: Should the Microbiota Be Taken into Account?

**DOI:** 10.3390/ijms25021165

**Published:** 2024-01-18

**Authors:** Andrea Galvan, Carlo Pellicciari, Laura Calderan

**Affiliations:** 1 Department of Neurosciences, Biomedicine and Movement Sciences, University of Verona, 37134 Verona, Italy; andrea.galvan@univr.it (A.G.); laura.calderan@univr.it (L.C.); 2 Department of Biology and Biotechnology, University of Pavia, Via A. Ferrata 9, 27100 Pavia, Italy

**Keywords:** human skin, in vitro models, skin microbiota, biological barriers

## Abstract

Skin plays crucial roles in the human body: besides protecting the organism from external threats, it acts as a thermal regulator, is responsible for the sense of touch, hosts microbial communities (the skin microbiota) involved in preventing the invasion of foreign pathogens, contains immunocompetent cells that maintain a healthy immunogenic/tolerogenic balance, and is a suitable route for drug administration. In the skin, four defense levels can be identified: besides the physical, chemical, and immune barriers that are inherent to the tissue, the skin microbiota (i.e., the numerous microorganisms living on the skin surface) provides an additional barrier. Studying the skin barrier function or the effects of drugs or cosmetic agents on human skin is a difficult task since snapshot evidence can only be obtained using bioptic samples where dynamic processes cannot properly be followed. To overcome these limitations, many different in vitro models of human skin have been developed that are characterized by diverse levels of complexity in terms of chemical, structural, and cellular composition. The aim of this review is to summarize and discuss the advantages and disadvantages of the different human skin models so far available and to underline how the insertion of a proper microbiota would positively impact an in vitro human skin model in an attempt to better mimic conditions in vivo.

## 1. Introduction

The “3Rs” (Replacement, Reduction, and Refinement) principle of humane animal research is accepted all over the world and is embedded in many national and international legislations [1]. The application of this principle has forced researchers in the biomedical field to reduce the number of animals employed in preclinical research and to find alternative in vitro models to obtain preliminary results on the suitability, safety, and efficacy of novel therapeutic agents. The predictivity level of the model is crucial since the obtained results will guide the following animal testing that is still needed to approach the clinical phase.

Among the tissues and organs that have been considered for reassembling in vitro, the skin represents an interesting and multifaceted example: it is the natural protecting covering of the body and the possible route for the administration of different local or systemic drugs, thus being of great interest in biomedicine and cosmetics [2,3]. The skin is composed of three layers: the most superficial epidermis, the dermis, and the hypodermis (or subcutaneous tissue), and also comprises several appendages found in the different strata (i.e., hair, sweat glands, and sebaceous glands that derive from the epidermis, the dermis and the upper part of the hypodermis) [3].

Many researchers describe the skin as the complex of four morphologically and functionally related barriers: the cell and tissue components are responsible for the physical, chemical, and immune skin barriers, while the skin microbiota, or dermobiota (i.e., the bacteria, fungi, viruses, archaea, and mites present on the skin surface) forms a complex ecosystem that keeps pathogenic bacteria under control and releases activating/modulating substances for the epidermal and immune cells, thus favoring the maintenance of the whole barrier functions [4,5]. In humans, skin microbiota is acquired at birth since, in utero, the skin is sterile; skin colonization occurs a few minutes after birth by the mother’s commensal microorganisms, and this process in the neonatal stage is crucial to establish immune tolerance towards them [6,7]. During the organism’s growth, the bacterial populations gradually adapt to the skin environment, and the microbiota colonization continues until equilibrium is reached in adulthood [8,9,10]. Serving as a physical barrier, the skin microbiota prevents the invasion of pathogens, but when the balance between commensals and pathogens is altered, the barrier may be broken, and skin diseases (and sometimes even systemic disorders) can occur [5].

**Figure 1:**
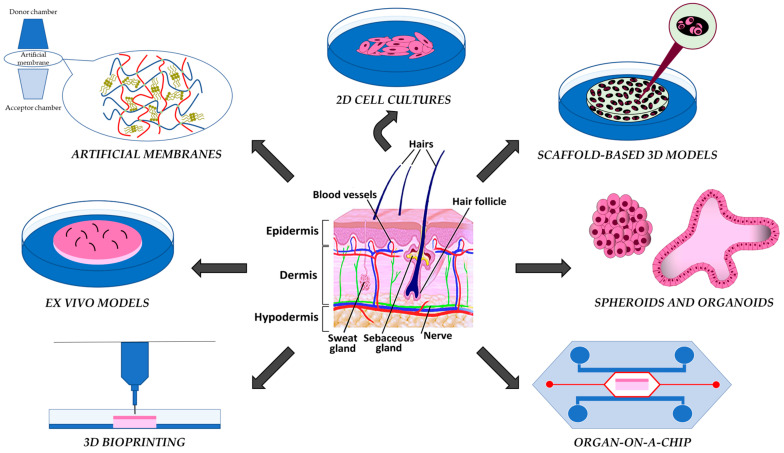
The in vitro models of human skin will be discussed.

The skin models in vitro have been designed to obtain reliable data on the biocompatibility, penetrability, safety, and efficacy of tested compounds, and their complexity has progressively improved to approach a reliable similarity to the native skin. From the simplistic polymeric membranes and two-dimensional (2D) cell monolayers, the skin models have been evolving into more complex and biologically relevant three-dimensional (3D) systems, where the skin organization is emulated in the presence or absence of scaffolding structures; furthermore, ex vivo skin fragments may be maintained in culture media under static or flow conditions (Figure 1).

In this review article, different experimental models of human skin will be described in order of complexity, and some of their applications will be presented, pointing out their advantages and disadvantages. In addition, it will highlight how the microbiota is an important component in an in vitro model to more correctly reproduce the structure and function of native human skin. The papers published over the last ten years have been considered, although the most relevant to the skin models in vitro were more recently issued (since 2018). The literature survey was made through the PubMed and Scopus databases, using “skin models”, “in vitro human skin” and “microbiota”, as search terms.

## 2. Artificial Membranes

The first cited models consist of artificially generated membranes: they are quite distant from the in vivo condition due to the lack of cells and intercellular matrix, but they can be useful for screening with respect to the chemical-physical characteristics of the compounds to be tested. Two main groups can be identified: the non-lipid-based and the lipid-based models [11].

In the first group, we find the microporous cellulose acetate (CA) membrane that is useful for studying the topical diffusion and release of chemical agents [12]. However, these systems have several disadvantages, e.g., the pore size of CA is larger than in human skin, thus causing a higher permeation rate than that found in the native biological barrier (in fact, de Almedida Borges and colleagues [13] observed a higher permeation of dapsone through CA membranes compared to the permeation observed in pig ear epidermis). In addition, CA membranes also lack the fluid lipid component, which is normally present in the stratum corneum of the in vivo skin and is one of the main barriers to drug penetration [12,13].

In order to overcome this limitation, lipid-based skin models have been developed, such as the skin PAMPAs (Parallel Artificial Membrane Permeability Assays), which contain free fatty acid, cholesterol, and synthetic ceramide analogs mimicking the stratum corneum [12,14]: the skin PAMPAs are 96-well plate-based systems where an artificial membrane is interposed between a donor and an acceptor compartment, in a sandwich-like structure. These systems attracted the attention of researchers as high-throughput assays for the preliminary screening and for obtaining permeation- and stability-related insights into topical and transdermal formulations [14,15]. For example, this model has been exploited by Rahma and colleagues [16] for the assessment of the permeation of phenoxyethanol, a common preservative ingredient in baby wipe formulations; notably, the authors observed a positive correlation for the total amount of permeated phenoxyethanol between this artificial membrane and porcine or human skin placed in a Franz diffusion cell, a widely used system to test drug permeation in vitro [16,17,18]. A skin PAMPA was also used to assess the penetration of retinol with the addition of natural jojoba oil as a penetration enhancer: interesting data were obtained on the transdermal delivery capability of jojoba oil since the retinol permeation registered was nearly 40-fold greater when associated with the oil, probably due to its ability to increase the fluidity of the lipid barrier [19]. However, skin PAMPAs have some drawbacks. In particular, they lack the stratified epidermal tissue and all the wide set of skin macromolecules, cell populations, and appendages, while the lipid mixture mimicking the stratum corneum does not contain all the lipid subclasses normally present in the human skin [9,10]. Luo et al. [20] performed a comparative study of ibuprofen permeation using human and porcine tissue, a skin PAMPA model, and a silicone membrane and found that the drug was more permeable through the PAMPA and the silicone membrane than through the animal tissues. Moreover, a skin PAMPA model was recently used to demonstrate the skin permeability of cannabidiol, but the authors themselves recognized the intrinsic limitations of the model used, suggesting that the preliminary results obtained might not reflect what would actually happen with human skin [21].

Strat-M membrane by EMD Millipore (Danvers, MA, USA) is a further example of lipid-based artificial membrane mimicking human skin; it is an inexpensive synthetic membrane composed of multiple layers of polyether sulfone (designed to have a very tightly packed surface simulating the stratum corneum) and more permeable underlying layer of polyolefin resembling the epidermis and dermis, to create a permeability gradient as it occurs in vivo [11,22,23,24]. Haq and her group [12] demonstrated in 2018 the superiority of Strat-M over the already mentioned CA membrane. In particular, the permeations of diclofenac sodium, hydrocortisone, and caffeine across the two different membranes were tested: it resulted that the permeability of Strat-M was closer to that of skin than that of CA membrane, presumably due to the larger pore size found in the latter; the CA membrane is also affected by a certain degree of permeation variability between replicates, which instead is negligible when using Strat-M. Besides this, in another study, Haq and co-workers demonstrated the good permeability correlation between Strat-M and human cadaver skin placed in Franz diffusion cells, testing different nicotine solutions containing penetration enhancers, highlighting the potential of the model [25]. Among the latest groups that used these artificial membranes, it is worth recalling Kittaneh’s group [22], who tested different film-forming solutions for the transdermal delivery of vitamin D3, and Czajkowska-Kośnik’s group [23], who instead used the Strat-M as a screening tool to predict the penetrability of nanostructured lipid carriers-based gel formulations for the delivery of etodolac.

All these artificial membranes can suitably be used for initially screening molecules or compounds, whose diffusion features must be more accurately investigated because these models do not adequately mimic the complex architecture and chemical composition of the natural skin; in particular, the cells are lacking, which limits the ability of these models to give insights about the safety, toxicity and potential therapeutic effects of the tested molecules. The passage to cell-based systems is thus necessary to obtain these data.

## 3. 2D and Scaffold-Based 3D Skin Models

2D cell-based models are important gold standard material to screen target molecules and test them for cytotoxicity or cellular uptake, although they have an intrinsic limitation due to the lack of the skin morpho-functional complexity.

Since their first investigation, Rheinwald and Green [26] observed that the growth of monolayers of human keratinocytes was supported by the co-culture with fibroblasts, being these cells able to secrete extracellular matrix (ECM) proteins and growth factors [3,13,26]. Many examples exist in the literature regarding the involvement of these easy-to-produce 2D cell cultures to evaluate the response of cells to a given stimulus. For example, Letsiou and her group [27] tested the effects of a potential ingredient for new personal care formulas, the sweet cherry (*Prunus avium* L.) extract, on 2D cell cultures of primary normal human epidermal keratinocytes, demonstrating that this compound is non-cytotoxic.

Besides primary cells that are directly obtained from living tissues and are characterized by a limited life span and stability in culture, immortalized cell lines that are able to survive with continuous cell division are suitable for 2D modeling [28,29,30]. As an example, spontaneously immortalized human keratinocytes (HaCaT cell line) were used by Smolińska et al. [31] to test the effects of isoflavone genistein on both normal and psoriasis-induced keratinocytes, to test the wide range of actions of this naturally occurring plant compound on both healthy and psoriatic epithelial cells. The results obtained suggest that this natural substance could ameliorate the aberrant gene expression condition found in psoriasis by down-regulating highly active genes and up-regulating poorly active genes in psoriatic cells.

Even if cell monolayers are a cheap and rapid experimental tool for identifying toxic compounds during the preclinical screening phases, the lack of the native 3D organization and the unnatural mechanical constraints to which cultured cells are subjected make these models poorly predictive, with an increasing risk of misleading results [3,32]. This is demonstrated by the investigation by Chen et al. [33], who compared the effects of silver nanoparticle administration to 2D monolayer keratinocyte systems and a 3D epidermal model: they found that the same doses of silver nanoparticles when administered to 2D cell cultures resulted in oxidative stress- and inflammation-related cytotoxicity, that did not occur with the 3D model.

To obtain 3D-grown cultures, scaffold-based techniques may be used, where the scaffold is intended as an analog for the ECM guiding the adhesion, growth, and differentiation of cultured cells to shape them in a given 3D configuration [34].

Producing functional 3D tissues is the goal of tissue engineering, which has two main fields of application: the first one aims at constructing new functional tissues/organs for transplantation, while the second one is the production of engineered tissues not to be used for patients, but as valid in vitro models for studying tissue physiology under healthy or pathological conditions [35].

When selecting a scaffold for tissue engineering, the natural architecture and properties of the native tissue should be kept in consideration. As already recalled, the scaffold must represent a surrogate of the stromal structure and the ECM, thus supporting cell adhesion, proliferation, and differentiation, as well as the production of the ECM itself, which, in the long term, will replace the original scaffold depending on its degradation and bioreabsorption [36]. Indeed, the scaffolding constructs are not intended as permanent implants but as a “first guide” or a template for tissue growth and are destined to be degraded into non-toxic materials by the newly formed tissue [37].

The choice of the scaffold material is a crucial step. Many different materials exist, and they can be divided into two main groups: those deriving from natural polymers and those from synthetic polymers, even if ceramic materials are preferentially used in bone tissue engineering, thanks to their high mechanical stiffness and rigidity [36,37]. Collagen, fibrin, and chitosan are among the natural polymers: they all have low toxicity, are biocompatible and biodegradable, and are biologically active so that the resulting scaffold actively interacts with the cells, generally promoting good cell adhesion and growth. Nevertheless, a major concern with these natural polymers is the batch-to-batch variability [36,37,38]. To solve this problem, synthetic biodegradable polymers like poly(lactic acid) and poly(glycolic acid) with a customized and reproducible architecture may be used as substitutes for the previously mentioned natural polymers; unfortunately, they are usually less biocompatible, are not bioactive, and their degradation results in a local pH decrease which may cause the necrosis of cells and matrix [36,37].

A point of strength of some of these 3D skin models is derived from an intuition that dates back to the early 80s of the last century when the importance of the air-liquid interface (ALI) as a key element for proper skin modeling was realized. It was indeed guessed that the ALI was crucial to obtain differentiated keratinocytes at the top of a multi-layered sheet of keratinocytes with the basal cell layer placed close to the culture medium, using de-epidermized dermis or collagen gel as support for their growth [39]. This aspect was also underlined in 2005 by Sun et al. [40], who validated the use of a bioreactor for the production of tissue-engineered skin and compared the characteristics of monocultures of endothelial cells, human dermal fibroblast and keratinocytes and a co-culture of these last two cell types when grown in submerged conditions or at the ALI. The last culture type further highlighted that establishing the ALI is a more advantageous approach than the immersion method. Indeed, in immersion, the cultures fail to cornify, and the keratinocytes cannot provide the normal barrier as in the living tissue, while the co-cultures of fibroblasts and keratinocytes showed an improved viability when exposed to the ALI. Frankart et al. [41] also demonstrated how the ALI is crucial using an original 3D model of the human epidermis obtained by seeding human skin-derived keratinocytes on polycarbonate filters. As reported by the same authors, the exposure of keratinocytes to an ALI enhanced the expression of genes essential in forming an effective epidermal barrier for protection against external threats. Thus, the presence of the stratum corneum in some 3D skin models, which is absent in all the 2D ones, is a great step forward in the reproduction of human skin in vitro.

Another type of scaffold may be obtained by tissue/organ decellularization. After the removal by cell lysis of the non-ECM components from the tissue and the removal of all the resulting nuclear and cytoplasmic debris, the scaffold obtained retains all the structural, mechanical, and biological properties of the original ECM, including growth factors, and results in a highly biocompatible system [38]. Here, it is obviously important to choose the proper and effective de-cellularization protocol, whose impact on the ECM architecture and composition should be as limited as possible. Even the following cellular seeding is a critical passage since it is possible to achieve an inhomogeneous distribution of cells into the scaffold [38]. For this reason, scaffold porosity is also a crucial aspect to take into consideration when developing a scaffold for 3D cell culture. The proper scaffold porosity provides available space for the seeded cells to grow, migrate, and interact, but it also allows fluids and nutrients to circulate effectively and, eventually, vascularization to occur [36].

In addition to the possibility of being used as clinical skin replacements and grafts, the skin bioengineered substitutes, such as the human skin equivalents (HSEs), can be exploited during preclinical and industrial research phases as models for drug permeability tests and toxicity screening [42,43,44]. Depending on the aim of the project, two different types of HSEs may be chosen: the reconstructed human epidermis (RHE), consisting of keratinocytes grown at the ALI, or the full-thickness HSE (FTHSE), where keratinocytes grow above a dermis-like structure containing fibroblasts [28,29,43].

Among the most popular and commercially available RHE models, EPISKIN^®^, EpiDerm™, and SkinEthic may be recalled [29,45]: they proved to be adequate to test the permeation and penetration of aqueous solutions, but also to assess the irritating potential of different tested compounds [45,46,47]. Besides these commercially available products, lab-made models were described in the literature, such as the one used by Muller and his group [48], which is based on normal adult human keratinocytes seeded in 12-mm-diameter polycarbonate Milli-cell-PCF insert with a 0.4 µm pore size. In particular, this RHE was used to test the residual antimicrobial activity after the topical application of chlorhexidine digluconate or octenidine dihydrochloride.

The FTHSEs better mimic the skin histology due to the presence of fibroblasts in the recreated-dermal compartment: the presence of fibroblasts proved to be important for the optimal proliferation and differentiation of keratinocytes, as they secrete mitogens (e.g., the keratinocyte growth factor, also known as fibroblast growth factor 7) and other soluble factors; on the other hand, keratinocytes release interleukin-1 (IL-1) that directly influence fibroblasts proliferation. The vital mutual interaction between these two cell types is thus re-established in FTHSEs [42], where the cell resistance to toxic compounds was also enhanced, thus making them more predictive for in vitro toxicological studies than the single-compartment models [42,43]. Epiderm FT™ and Phenion^®^ Full-Thickness Skin Model are commercial examples of FTHSEs. An application of the first one is described by Chadhuri et al. [49], who used it to test the skin care-related activities of bakuchiol (a meroterpene phenol sourced from the seeds of *Psoralea corylifolia* L.), confirming it as a functional analog of retinol. The Phenion^®^ Full Thickness Skin Model was selected for a very interesting and recent study by Rohrbeck et al. [50], where a psoriasis-like phenotype was induced by the addition of cytokines to test the healing effect of *Clostridium botulinum* C3 exoenzyme (C3bot): it was found that C3bot administration caused a reduction in the cytokine-induced psoriasis-like phenotype inhibiting the cytokine-induced expression of IL-6.

No doubt, these HSEs are suitable research tools, although they still differ from the native human skin as they do not contain all the original different cell populations, appendages, and vessels and may suffer from an altered barrier formation, which affects the predictivity of these models [51].

## 4. Scaffold-Free Models: Spheroids and Organoids

Spheroid culture represents the most common and simple technique for 3D cell culture, as it does not require external scaffolds to build up the 3D structure [52]. It is based on the natural tendency of adherent cells to aggregate, and it can be generated from a wide variety of cell types. Spheroids, also definable as microtissues, are produced from cultures of a single cell type or co-cultures (mono- or multicellular spheroids). They can be obtained: (a) using agitation-based methods, exploiting, for example, spinner flasks or bioreactors; (b) utilizing external forces like magnetic levitation; (c) using the liquid overlay technique, which suspends cell culture on non-adhesive surfaces promoting the interaction between cells, similar to the non-adhesive hydrogel microwell method; (d) using microfluidic platforms or (e) 3D bioprinting techniques or (f) the easier gravity-based hanging drop method [52,53,54,55,56]. By aggregating, cells can establish mutual contacts and produce specific microenvironments that allow them to express a tissue-like phenotype by the deposition of ECM proteins and to form nutrient, waste, and gas gradients [52,57].

A single cell culture was used by Aiello et al. [58] to investigate the effect of the dipeptide carnosine on the proteome of ultraviolet radiation type A irradiated human skin fibroblasts. In particular, primary dermal fibroblasts were utilized to generate the spheroids thanks to the hanging drop technique, and the obtained microtissue proved to be valid for demonstrating the protective action of this dipeptide against the harmful effects of ultraviolet radiation type A [58].

Thanks to the simple geometrical structure, cellular spheroids are widely used in biomedical studies, in particular for investigating growth, invasiveness, and metastasizing ability of solid tumors; indeed, multicellular spheroids are more and more considered essential research tools for testing the efficacy of potential treatments in cancer research [59]. The heterogeneity of solid tumors is indeed well reproduced by spheroids grown in vitro; they are able to emulate the effect of tumor microenvironment on drug transport, efficacy, and resistance thanks to their 3D structure, where it is possible to recreate also the necrosis and radiation-resistant hypoxic regions that are often present at the center of solid tumors [51]. One interesting example of spheroids in skin cancer research is reported by Vörsmann et al. [60]: the aim of this study was to prove that an organotypic full-skin equivalent harboring melanoma tumor spheroids can suitably mimic the cutaneous melanoma metastasis found in vivo. A metastatic cell line was selected and cultured to produce spheroids that were inserted into the dermal compartment of a 3D FTHSE to recapitulate the spatial organization and cellular complexity found in the organ-tumor crosstalk in vivo. The authors compared the effect of two different antitumor treatments on conventional melanoma cultures and the inserted melanoma spheroids and found that significant differences in the therapeutic outcome exist between regular 2D and the complex in vivo-like 3D models, further emphasizing that the simplistic 2D cell models may have poor predictability [60].

Nevertheless, the use of spheroids as skin equivalents also brings some drawbacks. First of all, the spheroids grow immersed in the culture media, which is a crucial limitation since the immersion prevents the formation of the ALI, which is necessary to simulate the epidermal differentiation as it occurs in the tissue in vivo [52]. Moreover, since cells are in direct contact with media, the blood vessel barrier normally found in the living tissue is not recreated, but also the lack of vascularization itself represents a limitation of this microtissue; even the difficulty in controlling spheroids geometry and size (usually between 65 and 300 µm when produced using spinner flasks) is a disadvantage of these systems increasing the variability of the derived data [54,56,61].

A great limitation of all the above-mentioned models is the lack of skin appendages, whose presence and function would help the in vitro model to better mimic the complexity of native human skin. Nevertheless, exploiting the potential deriving from stem cells, and in particular adult stem cells, pluripotent stem cells (PSCs), and embryonic stem cells, researchers have developed organoids 3D cultures trying to address this problem, and so recreating 3D appendage-bearing skin tissue [62,63]. With the proper stimuli, the mentioned stem cells can be guided in their differentiation in order to generate these in vitro models containing various cell types. The results are self-renewing and self-organizing culture systems capable of providing a highly similar reconstruction of the original tissue/organ. Such systems can be useful models for the study of organogenesis and developmental disorders, but also in the study of the early development of human skin and hair follicles; furthermore, they can be used as skin grafts or as useful models for the screening of new drugs [62,64,65,66,67,68,69]. Moreover, recently induced PCS (iPSC)-derived skin organoids have also been exploited for disease modeling [70]. An interesting example is presented by Ma et al. [71]: in this work, human iPSC-derived skin organoids with hair follicles and nerve cells were exploited to investigate the susceptibility to SARS-CoV-2 infection. The study was intended to elucidate the pathogenesis of hair loss in COVID-19 patients and to test the virus’s ability to attack neurons in the skin. The final results gave indeed interesting insights, like the downregulation of protein associated with the development of epidermal stem cells, the decreased proliferation ability of the epidermal cells around the hair follicles, and the confirmed attack of SARS-CoV-2 to skin neurons, pointing out the adequacy of the infection model developed in providing research guidelines for more in-depth future analyses [71].

Another interesting and in vivo-closer model is the one used by Jung and colleagues [72], who used a human iPSC-derived skin organoid as a platform to model atopic dermatitis (AD) by *Staphylococcus aureus* (*S*. *aureus*) colonization and infection, with the aim of elucidating the possibly direct role of *S*. *aureus* in causing AD. To do so, the authors managed to develop an in vivo-like skin organoid thanks to the activation of the Wingless-related integration site (WNT) signaling pathway, resulting in larger organoids with no off-target cartilage differentiation, and using an AIL culture method; they thus obtained skin organoids with stratified squamous epithelium, more closely resembling the adult human skin. Once again, the organoid-based model proved to be suitable for modeling disease and showed the structural damage to the skin barrier derived by *S*. *aureus* infection and the production of epithelial and dermal cell-derived cytokines, as well as the protective and therapeutic role of *Cutibacterium acnes* pre-treatment, as previously observed in AD patients [72].

However, despite the improvements made to the model by this group, the authors themselves recognize that the limits of skin organoids are still to be addressed. For instance, the absence of immune cells and other key cell types, and of the vascularization, with the consequent lack of proper nutrient and oxygen delivery and mechanical stimuli by the blood flow; therefore, in these systems, biochemical waste accumulates at the core level, which is detrimental for cell viability and results in the limited lifespan of organoids [61,62,64,72,73]. To address this problem, microfluidic devices have been developed and will be discussed later [61,73].

## 5. Organ-on-a-Chip

Among the latest and most advanced models, the so-called organs-on-a-chip must be cited. These systems are based on the microfluidic dynamic culture of cells inside micrometer-sized chambers, trying to recreate in vitro the physiology of a tissue or organ [74]. Given the microfluidic flow, the supply of nutrients and removal of waste products is more physiologically relevant, just like the mechanical stimuli derived from the fluid shear stress, which is important for cell growth and differentiation [74,75]. Besides this, further advantages of these models are provided by the small volumes of reagents needed, which reduces the costs and their high throughput capacity, making them particularly interesting, for example, during drug screening [74,76]. In particular, many different organs-on-a-chip exist, which differ from each other in several aspects. Among them, a discriminating element surely is the choice between the tissue to be transferred inside the chip or its own growth within it. In the first case, the tissue could be an explant from a donor or an HSE grown in vitro and then placed on the device [74]. The use of a skin biopsy would ensure the presence of all those cellular and structural components normally found in vivo, while the use of HSE would guarantee the presence of the different skin layers; nevertheless, as reported by Lee et al. [77], the transfer of a tissue previously grown in vitro outside the chip could bring variability in the results due to the potential inconsistency in the immobilization step. For this reason, the use of a chip able to host inside both the growth and differentiation of human skin resident cells could be more appropriate.

Among the in situ skin-on-a-chip, we can differentiate two groups of models. The first ones are based on a tissue grown in an open space inside the device, with a microfluidic channel passing underneath the tissue, while the second ones comprise those models where the channels are used also as compartments for culturing the tissue, with the intrinsic limitations in recreating the whole skin 3D structure [74]. These chips can be further classified into two main groups according to the system used to generate the flow of liquids inside the microchannels. In the first group, we can find all those models where the flow is obtained thanks to a gravity-driven system: indeed, the fluid movement is allowed thanks to the rotation or rocking of the system. Generally, the static environment is overcome by the gravity-dependent movement of culture fluid through the microchannels thanks to the rotational-reverse motion of the chip, held on a chip holder connected to an electric motor, as described by Song et al. [75] and Lee et al. [77]. An example regarding the use of this type of skin-on-a-chip model was recently reported by Kim et al. [78] in an article where the potential therapeutic ability of the microbial metabolite I3LA was tested on a skin-on-a-chip AD model, where the skin disease was recreated exploiting two AD-inducing factors, IL-4 and IL-13. The gravity-driven medium flow supported the HSE culture made up of human dermal fibroblasts and epidermal keratinocytes, which made it possible to identify I3LA as effective in suppressing AD in short-term use. Just one year before, the same author used basically the same pumpless skin-on-a-chip HSE model to test the effects of the antioxidant α-lipoic acid as an anti-aging agent; the results obtained showed that the α-lipoic acid administration helps skin growth and regulates diffusion of water and nutrients [79]. Even the antioxidant and anti-aging effects of coenzyme Q10 were tested on this kind of model by Kim et al. [78,79,80]. In all these investigations, keratinocytes were directly exposed to the air for differentiation. The gravity-based way to obtain the circulation of the fluid has some drawbacks. First of all, the stability of the flow rates is not so obvious since it may vary depending on the change in hydrostatic pressure over time; also, the possible non-uniform distribution of solutes and the difficulty establishing a firm air-epidermal interface are critical issues to be taken into account. Moreover, in these systems, the possibility of fresh media perfusion and waste removal is limited [81,82].

There is a second group of skin-on-a-chip models where the flow is obtained thanks to a pump, ensuring more effective and secure control of the flow rates of fluids for long periods [81]. Mori and his group [83] fabricated an original HSE with perfusable vascular channels coated with endothelial cells, these channels being connected at both edges to a culture device linked to a perfusion system made of a peristaltic pump responsible for the flow. An interesting recent example of a reconstructed human skin-on-a-chip under dynamic micropump-driven flow is presented by Vahav et al. [84]. With the aim of obtaining robust culture conditions, the authors developed an air-exposed epidermis on a fibroblast-populated hydrogel containing neopapillae spheroids cultured in dynamic conditions in a microfluidic bioreactor for 10 days, testing on it increasing concentrations of the skin sensitizer, cinnamaldehyde. It was found that cinnamaldehyde has dose-dependent cytotoxicity, and it was possible to detect in the microfluidics compartment the presence of different released cytokines (IL-18, specific for the contact sensitizer; the pro-inflammatory, Il-1β; the inflammatory, IL-23 and IFN-γ; and the anti-inflammatory, IL-10 and IL-12p70), which points to the great informational potential of the developed model [84]. The main drawback of this second group of skin-on-a-chip models is the tubing needed for their set-up, which is time-consuming to assemble and poses the potential risk of cross-contamination [81].

## 6. 3D Bioprinting

3D bioprinting is another innovative, state-of-the-art, and emerging technique whose applications fall into the preclinical and, potentially, the clinical field of plastic and reconstructive surgery. In the first case, this technology has been applied in the development of in vitro models, fabricating biological constructs by deposing precisely biomaterials, living cells, and different signaling molecules with hierarchical architecture resembling the one normally found in vivo; among the advantages, this procedure is highly automated and performed at high-throughput rates with a high test-retest reliability [85,86]. In the world of full-thickness human skin modeling, these advanced bio-fabrication technologies allow the development of the tissue pre-design and the following layer-by-layer assembly of cell-loaded bioprintable materials (the so-called bioinks) [87]. Basically, the cell types of interest and the appropriate bioink must be selected, and the 3D skin model must be designed. Then, the printing process starts, and after its completion, the skin equivalent may be used for the experiments and subjected to histological and molecular analysis [85].

Three different techniques are used to achieve this step. The laser-assisted technique prints cells with high activity and a high resolution despite the low throughput level; the inkjet-based technique, which can be divided into continuous inkjet printing and drop-on-demand printing, with the latter being more precise and wasting less bioink; and the extrusion-based bioprinting, characterized by a highly controllable printing but with a low resolution [88,89]. Actually, regardless of the technique chosen, the success of the procedure depends on the biomaterial and the cells used. The biomaterial, like synthetic polymers or natural polymers, should be “printable”, a property deriving from the rheology and cross-linking abilities of the material itself [90]. Different hydrogel materials like collagen, gelatin, fibrin, chitosan, and hyaluronic acid or their combination have been used as bioinks so far [85]. Usually, two types of cells are involved in these models, i.e., fibroblasts and keratinocytes, and no other cellular types are considered that are normally found in vivo. Actually, some exceptions exist. Indeed, Ng and his group [86] also incorporated melanocytes in their epidermal model to obtain a 3D bioprinted pigmented skin, while Baltazar’s group [91] generated a bioprinted vascularized skin tissue containing endothelial cells, fibroblasts, pericytes and keratinocytes. Actually, in this latter study, the tissue was produced as an artificial skin graft, which indeed was then implanted on the dorsum of the immunodeficient mice, and the presence of vascular cells within it triggered the revascularization of the graft itself, preventing necrosis. Vascularization is indeed one of the crucial limiting factors in skin bioprinting, as well as in most of the other skin models [90]. As a further progress in this technology, Liu et al. [92] produced a bioprinted human vascularized skin tissue of great interest: the protocol used in their study entails the presence in the dermal compartment of neonatal fibroblasts, pericytes, and human iPSC-derived endothelial cells and self-assembled blood vessels, which highlight the physiological relevance of the model. This technology promises to become a valid and routinely used platform to test cosmetic and topical products, and its potential was indeed recognized by companies such as L’Oreal, Natura Cosmetics, and Proctor Gamble that invested in their development [93,94].

The problem that should be overcome by this technology is the lack of bioprinted human skin of all the different cell types, nerves, and appendages that characterize the level of complexity of the original skin.

## 7. Ex Vivo Models

In the attempt to find alternative skin models mimicking the human stratum corneum for in vitro permeation studies, shed snake skin was proposed in the 1990s. In fact, there are few contradictory reports in the literature, where some authors concluded that “shed snake skin may be a useful model membrane for transdermal studies because of its similarities to human skin, ease of storage and handling, and low cost” [95] whereas others affirmed that “the conclusion to be drawn from this study is that shed snake skin cannot be considered to be a model membrane for in vitro studies as each species displays different permeation characteristics” [96], so that “whenever possible human skin should be used in absorption studies and not snake skin: otherwise, misleading results may be obtained” [97]. A more recent article [98] suggests that shed king cobra skin may be used as a barrier membrane for in vitro nicotine permeation studies.

To further refine the experimental skin model and make it more closely resemble organ physiology, researchers have resorted to keeping ex vivo skin explants in culture under vital conditions for as long as possible. These are often intermediate models between in vitro and in vivo experimentation and can have a high predictive capability, limiting animal experimentation if human skin is used. In fact, often the main limitation of current in vivo and ex vivo animal-derived models of skin are the intrinsic differences between the different species, making the animal models not so representative of the human skin environment [99,100,101], although interesting results have been obtained in permeation studies using porcine skin explants [102,103,104,105]. Certainly, ex vivo models are also very useful for screening compounds as they greatly accelerate the selection and characterization of the molecules, making ex vivo models a valid alternative approach for safety and efficacy testing. These new-generation models are useful tools to investigate the acute effects of locally applied molecules for therapeutic or cosmetic purposes [101,106,107]. Indeed, human skin biopsies from conventional or plastic surgery fully reproduce healthy or diseased skin morphology and physiology, and for these reasons, they are frequently used. Since the structure of the native skin is maintained, including cell populations and the dermal matrix, skin biopsies are the most appropriate material to investigate the effects of substances on human skin.

An example of ex vivo-based studies is the work by Sidgwick et al. [108], where different topical formulations containing green tea catechins were tested on human skin biopsies suspended in a static liquid culture medium: it was found that catechins administration for 7 and 10 days led to the downregulation of α-SMA, fibronectin, mast cell tryptase, mast cell chymase, TGF-β1, CTGF and PAI-1.

Besides keeping the biopsy with the dermal/hypodermic portion immersed in the medium solution added to a Petri dish or similar container, in literature, we can find protocols where the tissue is assembled in a Franz diffusion cell, as a support for skin permeation studies; in this model, which has effectively been used to accurately study percutaneous absorption kinetics, the concentration of the absorbed drug is measured in the receptor chamber filled up with a solution bathing the dermal face of the tissue [109,110]. Nowak and her group [111] used human abdominal skin from plastic surgery placed in the Franz diffusion cells to perform a penetration study of the ethanol-water extracts of fireweed (*Epilobium angustifolium*), showing that some phenolic acids in these extracts were able to penetrate the tissue and accumulated in it, exerting an antioxidant effect.

Other ex vivo-based models have also been developed that aim to reproduce the pathophysiological conditions of inflammation in explanted human skin [112,113]. Recently, our research group [113] proved the ability of an innovative fluid dynamic system to preserve human skin explants: this preservation device allowed better results if compared with the classic static preservation protocols, and it was possible to induce an inflammatory phenotype in these preserved skin explants through the administration of substance P and dithranol.

The ability of the explants to preserve the pathological features of affected skin and the potential of their use in the preclinical drug discovery research is well described, for example, by Tiirikainen et al. [114], who preserved the key immunopathological mechanisms of psoriasis in full-thickness biopsies of psoriatic skin. An interesting recent study used human skin explants to simulate and compare the phenomena related to burns versus those due to an inflammatory event in order to discriminate the earliest stages in the chain of physiological events under these different pathological situations [115].

Although they preserve the original 3D structure and contain all the constituents of human skin in vivo, even these systems are affected by some drawbacks: ethical permission is needed for sampling and processing, the biopsies may suffer from inter- and intra-sample variability, and the structural/functional preservation is limited in time [113].

## 8. The Four Levels of the Skin Barrier: A Focus on the Skin Microbiota

As already recalled, the skin has been described as consisting of four morphologically and functionally related barriers: the physical, chemical, immune, and microbiotic barriers that form an interactive network whose disruption may lead to infection, inflammation, allergy, or even cancer [4,5].

The chemical barrier (pH and lipids) maintains the moisture and the acid mantle of the skin, inhibiting the growth of bacterial pathogens as well as preserving the skin elasticity and waterproofing. A lipid skin barrier is made up of ceramides, free fatty acids, and cholesterol produced by the keratinocytes in the upper layer of the epidermis, the stratum corneum [116,117]. Further, keratinocytes establish the physical barrier that preserves the structural integrity of the skin by tight and adhesive intercellular junctions while carrying out immune functions, such as antigen presentation and the secretion of cytokines and antimicrobial peptides. An additional skin barrier level, the immune one, comprises innate and adaptive immune cells, which are either resident or recruited to the skin and sense danger signals, protect against pathogens, and exert memory responses. Nowadays, our view of the skin has evolved from a simply mechanical barrier to an active organ that can sense danger signals and mount perfectly adapted defense measures in response to invading pathogens [118,119,120,121].

In this view, the human skin microbiota is of paramount importance. Genomics-based investigation showed that a variety of different bacterial species coexist in the human skin microbiota, which, in contrast to the gut microbiota, is mostly made of Gram-positive Actinobacteria (e.g., *Cutibacterium* spp. and *Corynebacterium* spp.), including the Gram-positive *Staphylococcus* [122]. The array of microorganisms colonizing the epidermal surface is fairly stable and depends on the skin site (the so-called “biogeography” factor), with changes in the relative abundance of the bacterial taxa associated with moist-, dry- and sebum-rich microenvironments. For example, in healthy adult skin, sebaceous sites are predominantly colonized by the lipophilic *Propionibacterium* species, while the hydrophilic *Staphylococcus* spp. or *Corynebacterium* spp. are mostly abundant in moist regions, like the bends of the elbows and the feet [5,123].

Among the protective and homeostatic functions of the skin microbiota, a key role is played by the “colonization resistance”, indicating the protection against pathogenic bacteria due to competitive growth but also to the production of antimicrobial peptides (AMPs) [5]. For example, the natural cutaneous commensal, *Staphylococcus epidermidis* inhibits the colonization by the pathogenic *S. aureus* and induces the expression of AMPs, resulting in *S. epidermidis*-orchestrated innate immune alertness; additionally, it may enhance the epidermal barrier function stimulating the increased expression of the tight-junction proteins, occludin and ZO-1 [124,125]. Moreover, the microbe-secreted proteases are involved in the desquamation process and in the renewal of the stratum corneum, thus directly influencing the physical skin barrier. Just like bacteria, fungi like the *Malassezia* genus also show a protective behavior against potential pathogenic yeasts and mold colonization by producing a range of indoles [123]. From the chemical barrier point of view, some components of the skin microbiota have an important role in secreting lipase enzymes that hydrolyze free fatty acids from the triglycerides present in sebum, maintaining in this way a low skin pH, preserving the acid mantle, but also stimulating the skin immunity in producing β-defensin 2 [123,126]. Further innate factors modulated by the skin microbiota include IL-1α and components of complement C5alpha receptor; a downstream consequence of the effect of the skin microbiota on innate immunity is also an overall increase of adaptive immunity, pointing out the relationship existing between skin microbiota and immunity [126,127].

Thus, the efficacy of the epidermal barrier is the outcome of the crosstalk between the different skin barrier levels, with the microbiota contributing to the skin barrier from a physical, chemical, microbial, and innate/adaptive immunological point of view; these functions are guaranteed by a strictly-regulated and finely balanced microbiota [126]. Indeed, as a result of environmental, genetic, lifestyle-associated, hygienic, and immune system-related factors, the alteration of the microbial communities can lead to a pathological skin condition; it means that these factors can have an impact on ordinarily beneficial bacteria, making them disease-causing under certain conditions [5,128]. In an interesting review, Boxberger et al. [123] present an effective table showing the association between the modification of the skin microbiota and some dermatological pathologies. Among them, the relationship between acne vulgaris and *Cutibacterium acnes* (*Propionibacterium acnes*) is reported. Actually, the bacterium is a major commensal organism in the healthy skin flora residing in hair follicles and sebaceous glands, where it releases free fatty acids from the metabolized sebum triglycerides, thus performing a protective role against the invasion by pathogenic microbes [126,128]. Its pathogenic behavior does not start with the increased bacterial growth, but with the loss of diversity balance between *C. acnes* phenotypes and the following colonization of acne-associated strains enhanced by a hyper-seborrheic environment: indeed, the colonization of the skin by *C. acnes* increases in parallel with maturation of sebaceous glands during puberty [122,126,128,129]. Further examples of disease-microbiota association in human skin are AD (where the increased abundance of *S. aureus* in the lesions interferes with the host immunity, directly damaging the skin barrier), psoriasis (with abnormal colonization of *S. aureus* and decrease of *S. epidermidis* abundance in psoriatic lesions), and seborrheic dermatitis (where the most abundant fungal skin commensal, *Malassezia* is involved in the pathogenesis) [122,126,128,129].

## 9. Human Skin Microbiota on In Vitro Human Skin Models

The interaction between keratinocytes and microbiota is thus essential for the proper barrier function of the skin. For this reason, it is to be foreseen to improve human skin models by adding the complete microbiota to colonize the apical side of the model in order to get closer and closer to the in vivo condition [130,131,132].

In the literature, it is possible to find human skin models where single commensal species were added, but very few used the complete skin microbiota. The development of such a model is justified also by the inadequacy of the in vivo animal models in this sense. Indeed, besides having intrinsic morphological differences with human skin, disadvantaging germ-free animal models, rodent skin is characterized by its own microbiota, which obviously differs from the microbial communities of human skin; thus, the attempts to model human microbiota on animal skin are prevented due to the competition exerted by the native flora [133,134].

However, not even all the in vitro human skin models previously presented are suitable to host the human skin microbiota.

Synthetic membranes have an intrinsic limitation: the cells are not present in these models, and the different microorganisms could not establish any symbiotic relationship. Thus, in the literature, it is possible to find studies where these synthetic membranes are exploited more as a support to bacterial growth than as a reconstruction of an effective skin barrier. An example of this is the cellulose acetate membrane filter used by Janvier et al. [135] to test the effect of gaseous nitrogen dioxide on the human skin microbiota; in particular, the effects of this environmental pollutant were not tested on the complete microbiota, but on five bacterial strains isolated from the skin of healthy volunteers. Even the 2D models are not suitable for the development of systems capable of emulating the relationship between skin and microbiota. In fact, they lack the stratum corneum, and so the microorganisms would come in direct contact with proliferating keratinocytes and fibroblasts, which does not happen in vivo; moreover, these systems are disadvantaged by the faster growth rate of bacteria compared to human cells, preventing these systems from being maintained for more than 24 h [28,123]. Thus, the passage to 3D or ex vivo-based models is compulsory.

Loomis et al. [136] used an underdeveloped full-thickness EpiDerm and microbial isolates from swabs of healthy human skin to study the effects of individual taxa and a microbial community in the skin model. From the obtained results, the authors suggested a community effect in microbiome-host signaling since the effects obtained by the presence of the microbial community (i.e., changes in epidermal thickness, epidermal cell proliferation, and filaggrin production) were not entirely driven by any single microorganism. These results point out once again that the integration of the full skin microbiota is crucial in the human skin in vitro model. This aspect was also underlined by Landemaine et al. [137], who showed that the addition of a full skin microbiota collected from the inner forearm of a volunteer to a 3D skin model results in a stable number of bacteria up to 7 days, with a more proliferative epidermis showing a higher expression of proteins of desmosomes and tight junctions when compared to the same model but with the addition of just one bacterial strain (*S. epidermidis*): these results suggest the positive impact of the microbiota over the 3D skin model, promoting keratinocytes proliferation and cohesion.

The benefit of the in vitro skin model that would derive from the addition of the complete microbiota can also be seen from another point of view, that of personalized medicine. After pointing out that current skin-on-a-chips do not implement the microbiome, Fernandez-Carro et al. [138] underlined the value that would derive from the integration in these models of the particular microbiome of patients affected by conditions associated with microbiota dysfunction in order to develop personalized treatments. Talking about these skin-on-a-chips, the fact that they do not fully recreate the in vivo conditions may be the reason that justifies the limited number of studies performed on host and skin microbiota in microfluidic devices [139].

However, most of the skin models used nowadays for the study of barrier functions lack skin appendages such as sebaceous glands and hair follicles; as a consequence, it is difficult or even impossible to co-culture bacteria strains that grow within anaerobic environments like *Cutibacterium*, limiting in this way the culturing of a complete human skin microbiota [136]. Thus, the development of skin appendages-containing human skin models would be important.

An interesting study was performed by van Der Krieken et al. [140], who planted the complete microbiota taken from the back of healthy volunteers onto an originally developed simplified human skin model, which consisted in the reproduction of the stratum corneum only by using human callus on top of agar in phosphate-buffered saline. Since in vivo skin bacteria live attached to the dead corneocytes, the stratum corneum was intended as a substrate and source of nutrients for the microorganisms. Indeed, the model was suitable for the growth of the most relevant strains but also for the complete set of skin microbes that are normally found in vivo, which remained relatively stable for a period of 7 days [140]. This model is clearly interesting due to its simplicity, but its intrinsic limitation as a model to study skin permeation or in recreating the full tissue complexity is obvious since only the outermost skin layer is recreated, thus excluding all the other components of the multilayered tissue.

Therefore, the use of human skin explants is certainly more suitable, but the systemic pre-surgical prophylaxis normally performed before surgical operations may affect the integrity of the native microbiome, a problem that can only be overcome by using skin explants deriving from non-prophylaxed donors [133].

## 10. Concluding Remarks

The development of in vitro systems mimicking as much as possible the structure, function, and behavior of a specific organ/tissue is fundamental to obtaining highly predictive preclinical data in order to minimize the number of animals involved in the study before moving to the clinical phase. Among these systems, in vitro models recreating the complex architecture of human skin are certainly useful in the study of the permeation, safety, and toxicological aspects of new potential formulations. Indeed, the skin is a formidable barrier protecting the organism from external threats, which are physical, chemical, and microbial in nature, and hinders the entry of topically applied substances intended to have a therapeutic or cosmetic action [141].

**Figure 2:**
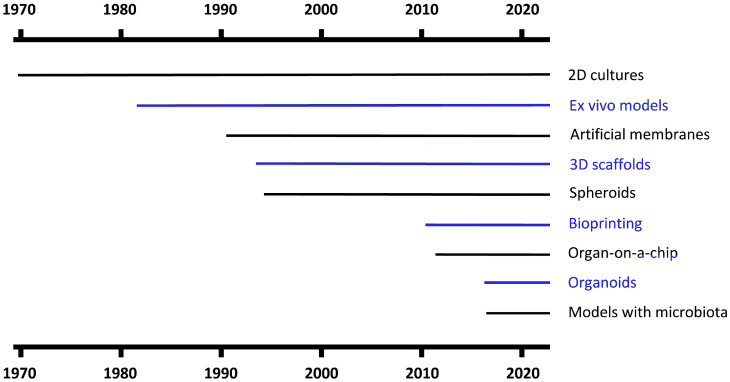
Timeline of the skin models considered, since the first report in the literature proposing their application.

Different skin models were proposed and evolved in time since the 1970s (Figure 2) and are presently characterized by an increasing level of complexity and fidelity to the in vivo situation, ensuring in this way an always better representation of the biological membrane in the lab. However, even if the disadvantages of human skin in vitro models have been pointed out, it should be underlined that all of them did play and are still playing a fundamental role in the research world.

Besides the integration of all the different skin layers, cellular populations, appendages, etc., the complete human skin microbiota should not be forgotten. The symbiotic relationship existing between the tissue and the microorganisms living on its surface underlines the importance of integrating them in these in vitro systems, obtaining in this way positive effects on the growth and cohesion of skin cells, which are then reflected on an improved barrier function. Thus, to better mimic the human skin, these models should also be able to host the complete microbiota communities: to do so, the presence of skin appendages like sebaceous glands is important for all those skin-related microbes living in these anaerobic environments. The use of human skin explants could solve all these issues, but the preservation after the explant, the difficulty in finding them, the inter-subject variability, and the possible alteration of the microbiota due to pre-surgical prophylaxis could limit their use.

The research must make further efforts to develop systems increasingly closer to the in vivo situation, something that does not seem so impossible given the extraordinary progress that has occurred in recent years.

It is easy to understand that the same complexity found in this organ should be present in the chosen model for a correct evaluation of the formulation permeability since both a transepidermal and a transappendageal pathway exist [142]. Moreover, the presence of the diversified cellular populations and neurovascular and stromal organization normally present in the tissue are essential to ensure a more in vivo-like situation. Therefore, researchers should focus on the development of in vitro models bearing all the constituents normally found in vivo, including the skin microbiota.

## Data Availability

No new data were created or analyzed in this study. Data sharing is not applicable to this article.
